# A Case of Refractory Childhood Glaucoma Associated With Sturge–Weber Syndrome Treated With Baerveldt Glaucoma Implant

**DOI:** 10.1155/crop/8624998

**Published:** 2026-01-08

**Authors:** Wakako Imamura, Akira Matsuda, Akira Hirota, Kohei Kuroda, Yorihisa Kitagawa, Tadashiro Saeki, Tomoka Kambe, Shutaro Yamamoto, Satoru Yamagami

**Affiliations:** ^1^ Division of Ophthalmology, Nihon University School of Medicine, Tokyo, Japan, nihon-u.ac.jp; ^2^ Department of Ophthalmology, Saitama Children′s Medical Center, Saitama, Japan, saitama-med.ac.jp; ^3^ Department of Ophthalmology, Juntendo University School of Medicine, Tokyo, Japan, juntendo.ac.jp

**Keywords:** Baerveldt glaucoma implant, childhood glaucoma, diffuse choroidal hemangioma, postoperative hypotony, Sturge–Weber syndrome

## Abstract

We reported the clinical course of refractory childhood glaucoma associated with Sturge–Weber syndrome (SWS) treated with Baerveldt glaucoma implant (BGI). The patient was a 14‐year‐old male diagnosed with SWS after birth. He had previously undergone four trabeculotomies for glaucoma and vitrectomy for submacular hemorrhage from a diffuse choroidal hemangioma in his left eye. Before the BGI surgery, his intraocular pressure was 30 mmHg under full medications. The patient experienced extensive serous retinal and choroidal detachments on Postoperative Day 9. The cause may have been the increased leakage of serous fluid from the choroidal hemangioma and the high venous pressure in the episclera due to SWS. Retinal and choroidal detachments subsided within 8 days with conservative therapy. It is important to avoid postoperative hypotony for the treatment of secondary childhood glaucoma due to SWS.

## 1. Introduction

Sturge–Weber syndrome (SWS) is a neurocutaneous syndrome with a triad of capillary–venous malformations in leptomeninges, facial hemangiomas in the trigeminal region, and glaucoma that occurs at a rate of 1 per 20,000 to 50,000 live births. [1]. Recent report showed that a somatic mosaic mutation in the GNAQ gene was associated with the development of SWS [2]. Glaucoma associated with SWS is diagnosed in infancy in 60% of cases [3]. Impaired aqueous humor outflow due to abnormal iridocorneal development was observed in early‐onset secondary glaucoma due to SWS. In such cases, gonioscopy examination may show high iris insertion, which is also observed in primary congenital glaucoma. On the other hand, the remaining approximately 40% of glaucoma cases are diagnosed later in childhood. The cause of these late‐onset secondary glaucoma due to SWS is thought to be the involvement of elevated episcleral venous pressure (EVP) in addition to abnormal angle development, which can lead to increased flow resistance distal to the Schlemm′s canal. Secondary glaucoma due to SWS often requires surgery; however, it is known that there is significant risk of hypotony‐related complications. Previous reports showed that existence of choroidal hemangioma is a risk factor for postoperative choroidal detachment and hemorrhage due to expansion of hemangioma with effusion of fluid into the suprachoroidal space[4]. In this report, we present a case of refractory secondary glaucoma due to SWS with choroidal hemangioma, which was treated with a Baerveldt glaucoma implant (BGI) surgery.

## 2. Case Report

A teenage boy with glaucoma due to SWS was referred to Nihon University Itabashi Hospital. Port‐wine nevus was observed on the left face after birth. Shortly after birth, he was diagnosed with secondary childhood glaucoma due to SWS. He experienced three trabeculotomy (TLO) surgeries for glaucoma of his left eye. Due to traumatic submacular hemorrhage from diffuse choroidal hemangioma, a vitrectomy with submacular tissue plasminogen activator (tPA) injection was performed for his left eye after third TLO surgery (Figure 1). Due to reelevation of the intraocular pressure (IOP), a fourth TLO surgery for his left eye was performed. Thereafter, the IOP gradually increased despite the use of latanoprost drops, brimonidine drops, and oral acetazolamide 500 mg daily. At initial examination his IOP was 30 mmHg in the left eye, iris adhesions at 6 and 12 o′clock and hemangioma of the superior episclera (Figure 2) were observed. BGI (BG103–250 mm^2^) was inserted under the superior and external rectus muscles, and the tube was completely ligated with two 8‐0 Vicryl sutures. The tip of the tube was inserted into the anterior chamber after injection of viscoelastic material. For the risk of postoperative hypotony, no slits were placed on the tube. The surgery was completed by covering the exposed area of the tube with a preserved sclera and suturing the conjunctiva, with viscoelastic material left in the anterior chamber. The IOP of his left eye was 25 mmHg the day after surgery. Acetazolamide oral administration of 500 mg, latanoprost, and brimonidine eye drops were continued and dexamethasone eye drops were started. The patient was discharged on the fourth postoperative day without any adverse events, and the IOP of his left eye was 28 mmHg. On Postoperative Day 8, the patient noticed deterioration of visual function without any ocular pain. On Postoperative Day 9 examination, the anterior chamber was shallow, and the retina and the choroid were visible behind the lens (Figure 3a). We diagnosed his left eye with serous choroidal and retinal detachments (Figure 3b). B‐scan ultrasonography did not reveal a high‐density area (Figure 3c), which suggested choroidal hemorrhage. Therefore, we considered this elevation (Figure 3a,b) as serous choroidal and retinal detachments. The IOP of his left eye was 14 mmHg. All antiglaucoma medications were discontinued, and 1% atropine eye drops were started with dexamethasone eye drops. Thereafter, the choroidal and retinal detachments gradually improved and were almost completely absorbed on Postoperative Day 17 (Figure 4a,b). After tube liberation around Day 34, IOP decreased to 10 mmHg and mild choroidal and retinal detachments reoccurred (Figure 5a,b), and then completely resolved on Day 125 after surgery (Figure 5c). His left eye remained in a stable condition thereafter; IOP has been maintained around 12–14 mmHg.

Figure 1Near‐infrared reflectance image (a), optical coherence tomography image (b), and fundus photograph (c) of submacular hemorrhage. Fundus photograph of the right eye (d) and the left eye (e). Submacular hemorrhage has been cleared in the left eye at 6 months after vitrectomy. Note the difference of the choroidal color of both eyes.

**Figure:**
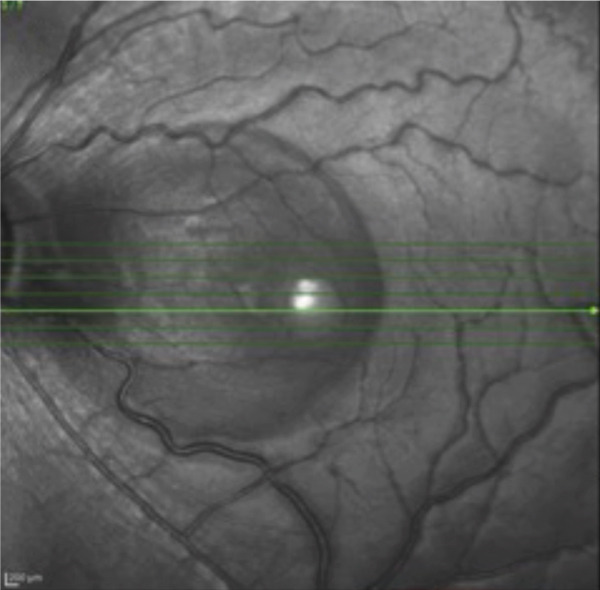
(a)

**Figure:**
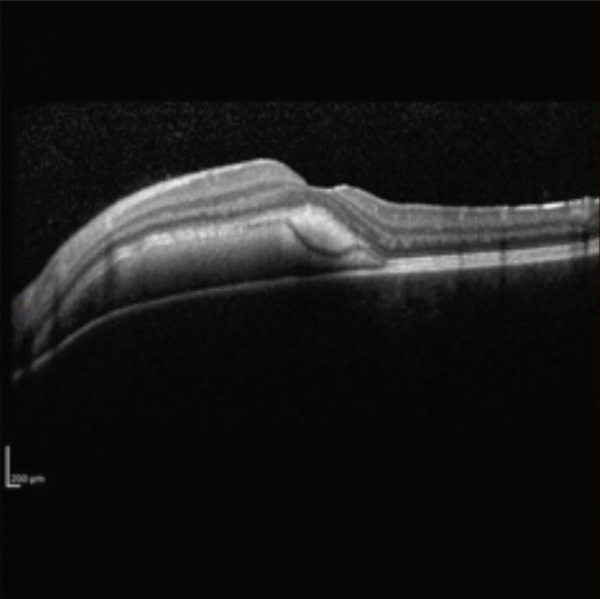
(b)

**Figure:**
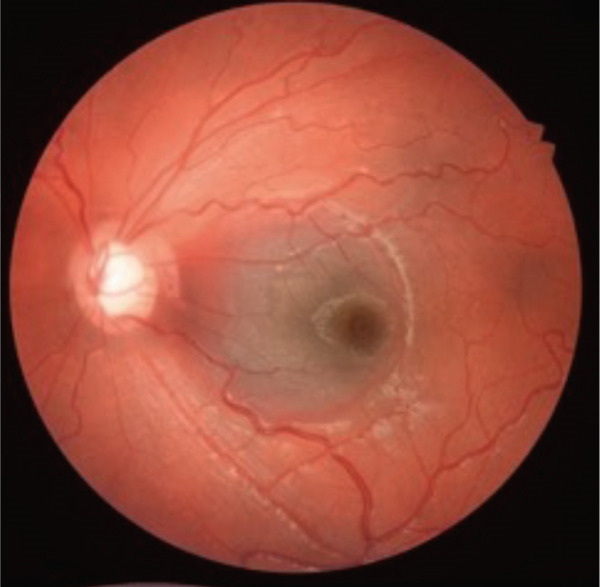
(c)

**Figure:**
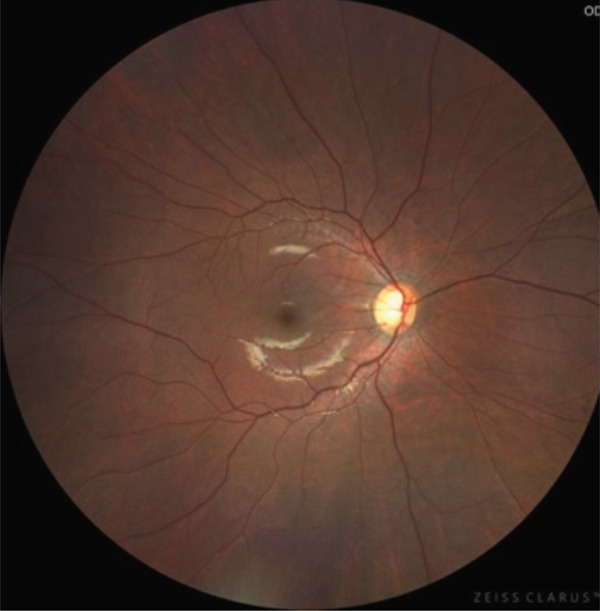
(d)

**Figure:**
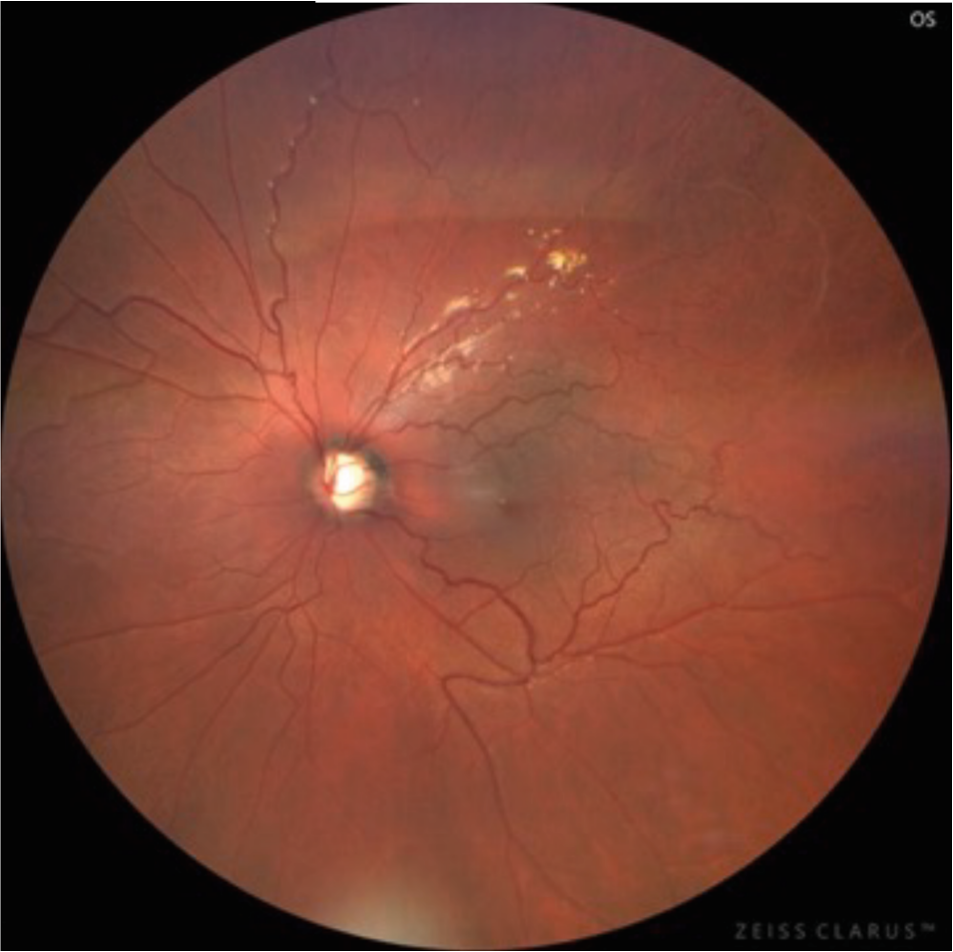
(e)

Figure 2Slitlamp photographs (a, b), Humphrey 24‐2 visual field examination (c), and fundus photograph (d) of the patient′s left eye at preoperative examination. Iris atrophy due to previous TLO surgeries was observed (a), and episcleral hemangioma was also observed (b). Central scotoma (c) and glaucomatous optic disc cupping (d) were observed. Tortuosity of the retinal vessel (arrowhead) was also observed.

**Figure:**
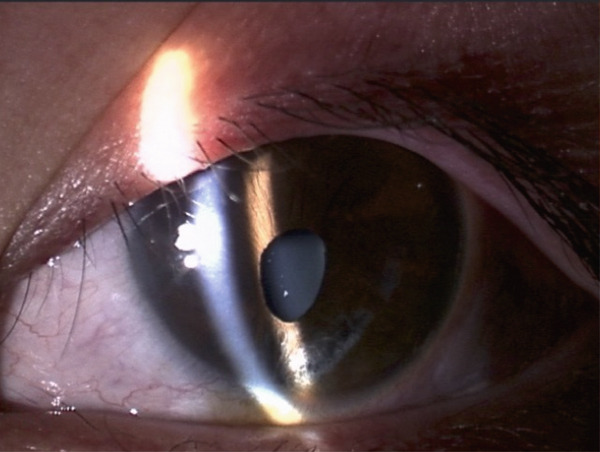
(a)

**Figure:**
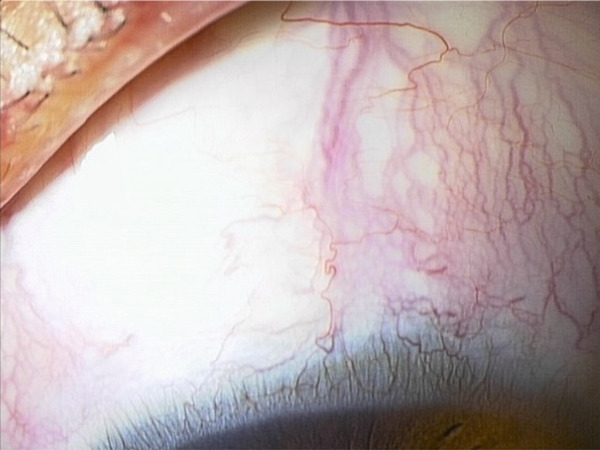
(b)

**Figure:**
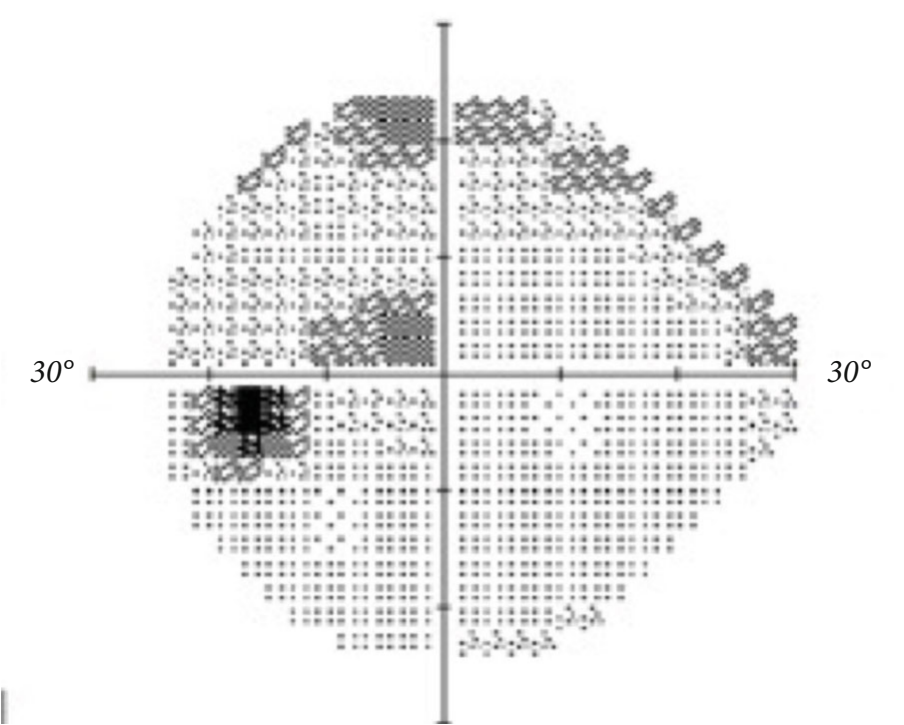
(c)

**Figure:**
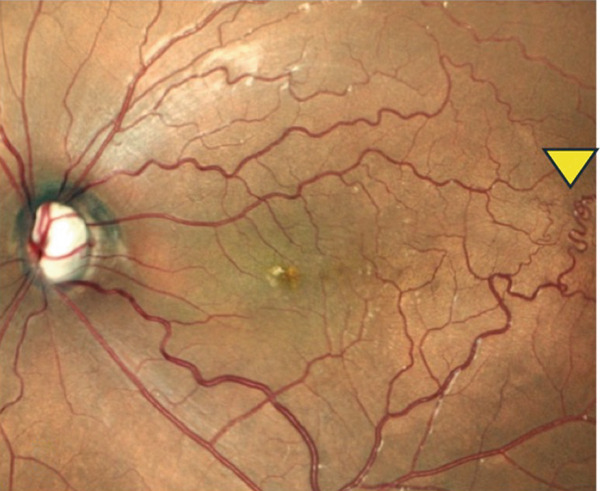
(d)

Figure 3Slitlamp photograph (a), fundus photograph (b), and B‐scan ultrasonography (c) of the patient′s left eye on Postoperative Day 9. Note retinal surface at the vicinity of lens with demarcation line (arrows) between retinal and choroidal detachments (a) and the extensive retinal and choroidal detachments (b, c).

**Figure:**
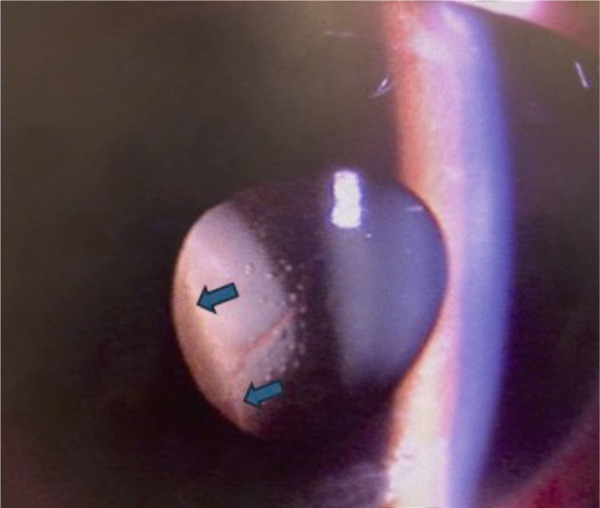
(a)

**Figure:**
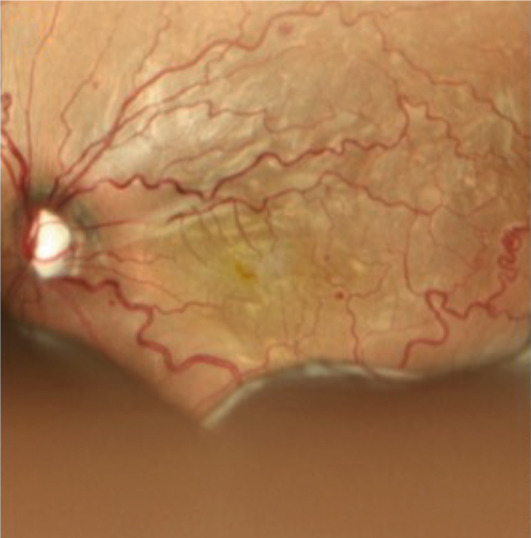
(b)

**Figure:**
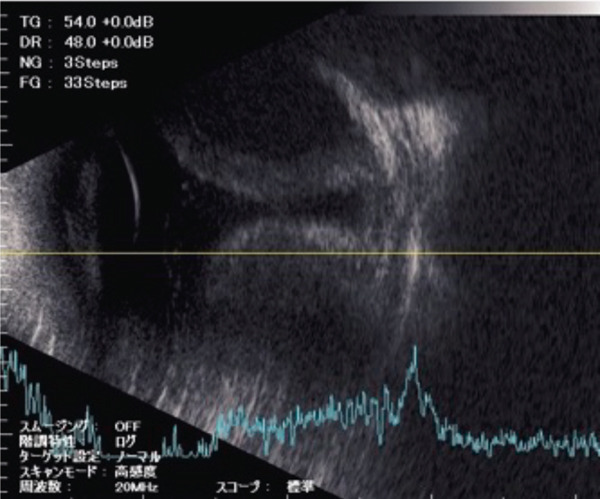
(c)

Figure 4Fundus photograph (a) and B‐scan ultrasonography (b) of the patient′s left eye at Postoperative Day 17. Almost complete resolution of the retinal and choroidal detachments was observed.

**Figure:**
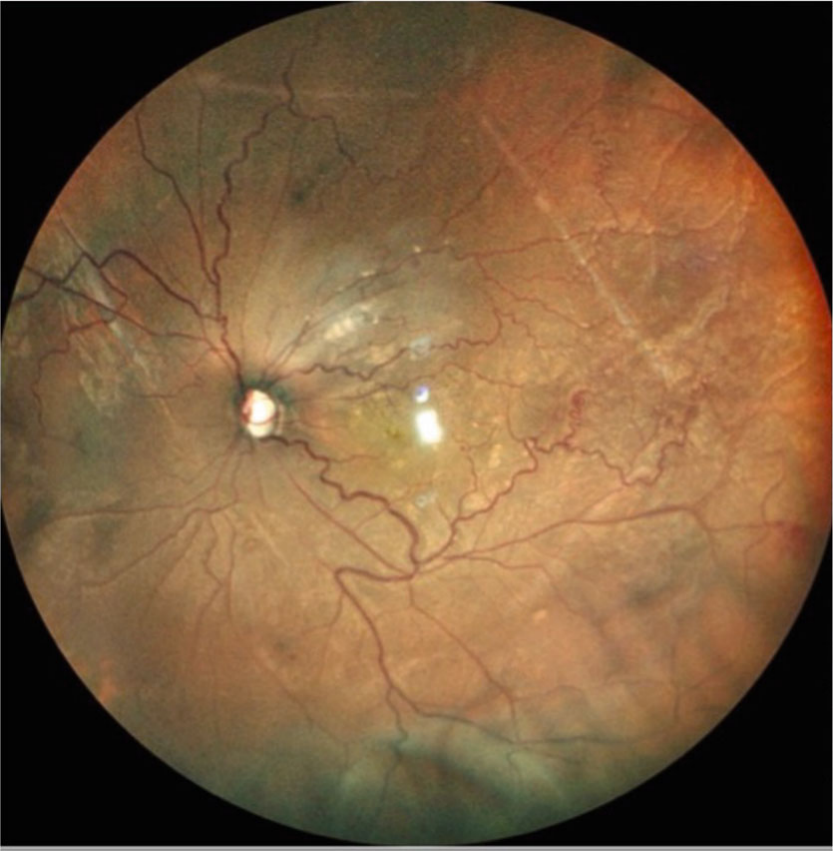
(a)

**Figure:**
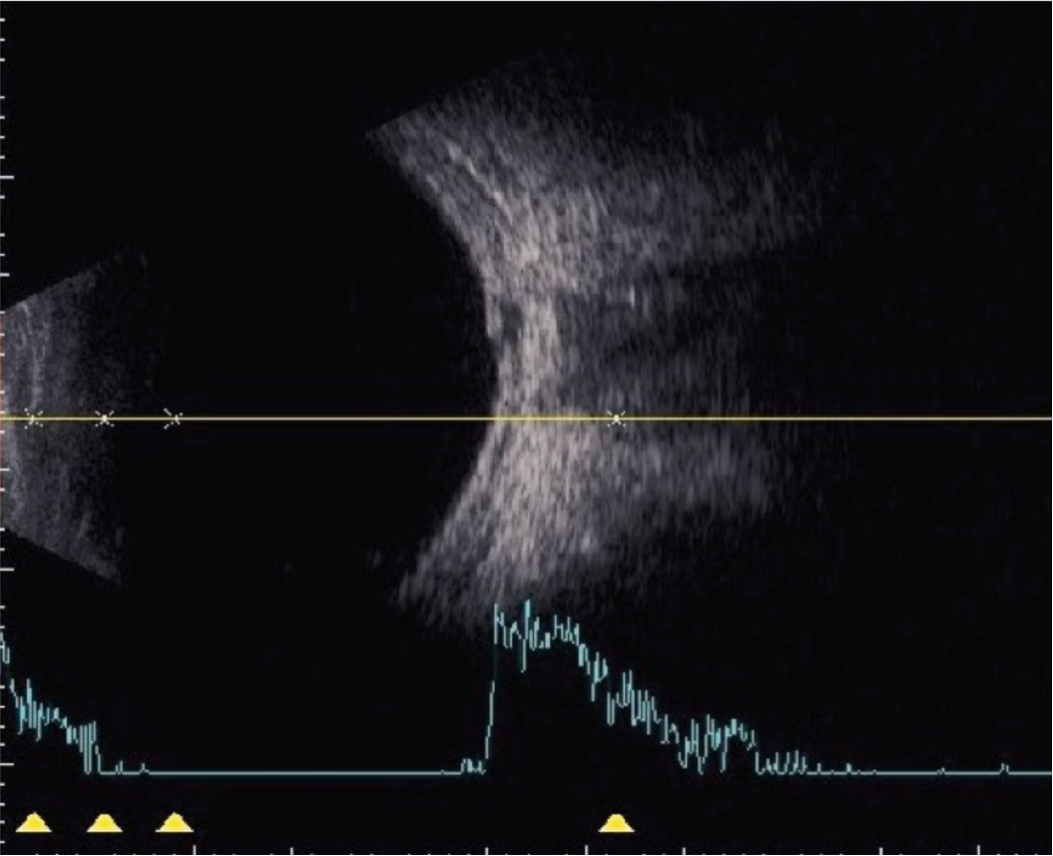
(b)

Figure 5Chronological change in IOP during postoperative period (a), arrows indicate Day 9 (first choroidal detachment) and Day 34 (recurrence). Fundus photographs at Postoperative Day 34 showing recurrence of retinal and choroidal detachments (b) and at Postoperative Day 125 showing reabsorption (c).

**Figure:**
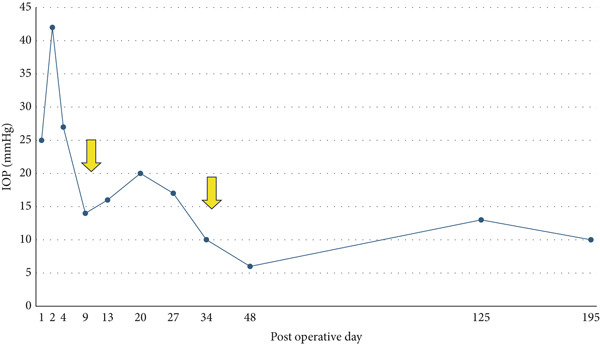
(a)

**Figure:**
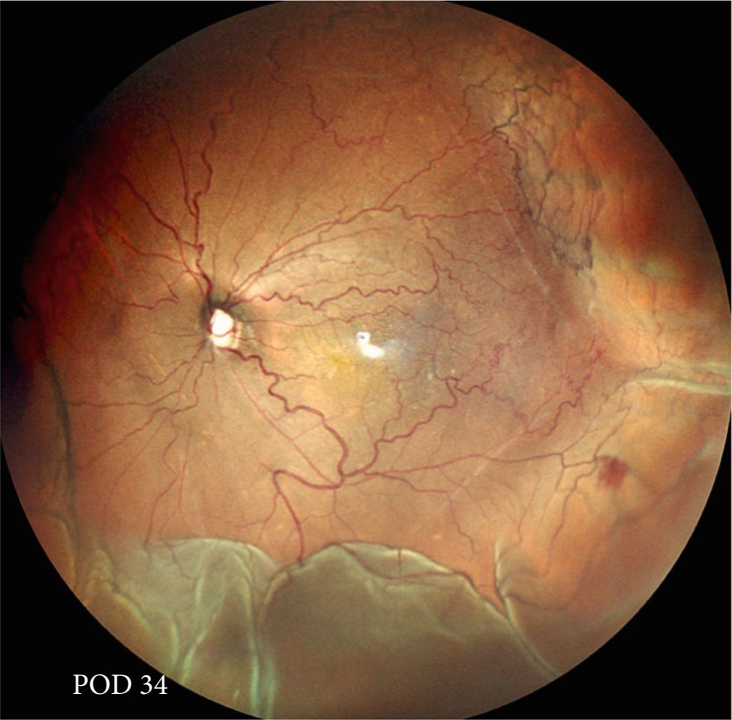
(b)

**Figure:**
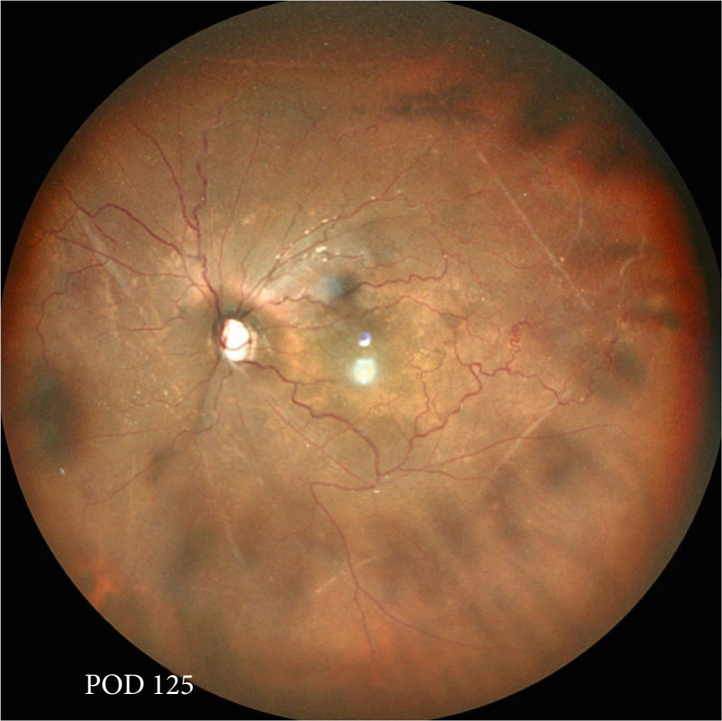
(c)

## 3. Discussion

We presented a case of childhood secondary glaucoma with SWS diagnosed in his infancy. Repetition of TLO surgeries contributed to maintaining IOP; however, there was no remaining trabecular meshwork to be opened after the fourth TLO. We selected filtration surgery for his treatment because the main outflow resistance seems to be distal to Schlemm′s canal. Filtration surgery for SWS requires caution because of the high incidence of choroidal detachment, hemorrhage due to the sudden decrease in IOP [5]. It was reported that the existence of choroidal hemangioma is a risk factor for postoperative choroidal detachment [4], and choroidal hemangioma exists in his left eye (Figures 1 and S1). Therefore, we consider that there was a significant risk of choroidal detachment and/or hemorrhage for his left eye. We selected BGI rather than trabeculectomy (Trab) for the refractory glaucoma secondary to SWS because moderate scarring of conjunctiva due to previous surgical manipulation existed. Moreover, it is possible to minimize sudden reduction of IOP using complete ligations of the tube in the case of BGI. One previous report showed the results of BGI insertion for the treatment of secondary childhood glaucoma due to SWS, in which five eyes were treated successfully (IOP < 21 mmHg, median follow‐up of 87 months) using BGI and one eye failed due to high IOP [6]. Another report showed the results of Ahmed glaucoma valve (AGV) implantation and Trab for 40 eyes of secondary childhood glaucoma due to SWS [4]. According to the report, IOP control success (< 21 mmHg) rate was 80% for AGV (20 eyes, average 23.15‐month observational period) and 70% for Trab (20 eyes, average 22.95‐month observational period) with two eyes (AGV) and one eye (Trab) of postoperative choroidal detachment. Considering the better long‐term IOP control ability of BGI rather than AGV [7], we selected BGI for this case. The patient had a temporarily high IOP (25–28 mmHg) after surgery due to complete ligation of the tube and remaining viscoelastic material in the anterior chamber. It was reported that the arteriolar pressure is directly transferred to the choroidal vascular space without being reduced by a capillary bed; the choroidal intravascular pressure would be around 30 mmHg in the case of SWS with choroidal hemangioma as in this case [8]. His IOP decreased gradually from the Postoperative Day 4 (28 mmHg) to Day 8 (14 mmHg), and it caused the expansion of the choroidal vascular component and subsequent leakage of fluid, resulting in extensive choroidal detachment (Figure 3). Although the exact cause of the choroidal detachment is unknown, in addition to increased leakage of serous components from the choroidal hemangioma, the high EVP associated with SWS and the absence of vitreous due to prior vitreous surgery may play some roles in the aggravation of his choroidal detachment. We considered the possibility of early tube release at this point (Day 9) to be unlikely because his IOP was maintained at 14 mmHg and there was no clear bleb formation above the plate, so we decided to treat conservatively by discontinuing antiglaucoma medication and adding atropine eye drops rather than reoperation. Considering the high risk of choroidal detachment in this case, it might have been better to taper antiglaucoma medication earlier to prevent or minimize choroidal detachment. The selection of BGI as a method of filtration surgery seems to be appropriate because IOP was retained in the middle teens despite the extensive choroidal detachment, and at the time of tube release (around 34 days), fibrotic membrane around the plate was already formed; therefore, the second wave of choroidal detachment was mild (Figure 5b) and did not affect the clinical course of his treatment.

To prevent postoperative hypotony, the tube was completely tied with 8‐0 Vicryl in this case. Significant choroidal detachment was observed at the 9th postoperative day, when the IOP was 14 mmHg. The IOP of 14 and 10 mmHg, which induced choroidal detachment in this case, cannot necessarily be considered extremely low IOP. One possible cause of choroidal detachment despite the absence of excessively low IOP includes the high EVP of SWS and the presence of abnormal vascular permeability in the vascular network due to concomitant choroidal hemangioma. In conclusion, we demonstrated that tube shunt surgery using BGI‐250 was effective for secondary pediatric glaucoma with SWS and concomitant choroidal hemangioma that had not responded to multiple glaucoma surgeries. Although choroidal detachment could not be prevented despite complete tube ligations, the condition improved with conservative treatment without the need for further surgery. We hope this case will serve as a reference for the treatment of similar cases in the future.

## Conflicts of Interest

The authors declare no conflicts of interest.

## Funding

This study was supported by the JSPS KAKENHI with Grant Number JP22K09819 to A.M. (JP22K09819) and by the Hoya Foundation to A.M.

## Supporting information


**Supporting Information 1** Additional supporting information can be found online in the Supporting Information section. Figure S1: OCT image of his right eye (OD) shows normal choroidal vascular pattern, whereas his left eye (OS) shows increased choroidal thickness and loss of choroidal vascular pattern.

## Data Availability

The data that support the findings of this study are available on request from the corresponding author. The data are not publicly available due to privacy or ethical restrictions.

## References

[1] Comi A. M. , Update on Sturge-Weber Syndrome: Diagnosis, Treatment, Quantitative Measures, and Controversies, Lymphatic Research and Biology. (2007) 5, no. 4, 257–264, 10.1089/lrb.2007.1016, 2-s2.0-38149078260, 18370916. 18370916

[2] Shirley M. D. , Tang H. , Gallione C. J. , Baugher J. D. , Frelin L. P. , Cohen B. , North P. E. , Marchuk D. A. , Comi A. M. , and Pevsner J. , Sturge-Weber Syndrome and Port-Wine Stains Caused by Somatic Mutation in GNAQ, New England Journal of Medicine. (2013) 368, no. 21, 1971–1979, 10.1056/NEJMoa1213507, 2-s2.0-84877957142, 23656586. 23656586 PMC3749068

[3] Mantelli F. , Bruscolini A. , La Cava M. , Abdolrahimzadeh S. , and Lambiase A. , Ocular Manifestations of Sturge-Weber Syndrome: Pathogenesis, Diagnosis, and Management, Clinical Ophthalmology. (2016) 10, 871–878, 10.2147/OPTH.S101963, 2-s2.0-84968906525, 27257371. 27257371 PMC4874637

[4] De Sarker B. K. , Malek M. I. , Mannaf S. M. , Iftekhar Q. S. , Mahatma M. , Sarkar M. K. , and Rahman M. , Outcome of Trabeculectomy Versus Ahmed Glaucoma Valve Implantation in the Surgical Management of Glaucoma in Patients With Sturge-Weber Syndrome, British Journal of Ophthalmology. (2021) 105, no. 11, 1561–1565, 10.1136/bjophthalmol-2020-317098, 32912851. 32912851

[5] Javaid U. , Ali M. H. , Jamal S. , and Butt N. H. , Pathophysiology, Diagnosis, and Management of Glaucoma Associated With Sturge-Weber Syndrome, International Ophthalmology. (2018) 38, no. 1, 409–416, 10.1007/s10792-016-0412-3, 2-s2.0-85008474100, 28064423. 28064423

[6] Karaconji T. , Ting E. R. , Zagora S. L. , Ardern-Holmes S. , Jamieson R. V. , and Grigg J. R. , Surgical Treatment for SWS Glaucoma: Experience From a Tertiary Referral Pediatric Hospital, Journal of Glaucoma. (2020) 29, no. 12, 1132–1137, 10.1097/IJG.0000000000001645, 32852376. 32852376

[7] Badawi A. H. , Al Owaifeer A. M. , Mofti A. , Al-Shahwan S. , Jadaan A.I, and Malik R. , Comparison of the Ahmed and Baerveldt Glaucoma Drainage Implants in the Treatment of Primary Congenital Glaucoma, Journal of Pediatric Ophthalmology and Strabismus. (2023) 60, no. 6, 448–454, 10.3928/01913913-20230119-02, 36803242. 36803242

[8] Iwach A. G. , Hoskins H. D.Jr., Hetherington J.Jr., and Shaffer R. N. , Analysis of Surgical and Medical Management of Glaucoma in Sturge-Weber Syndrome, Ophthalmology. (1990) 97, no. 7, 904–909, 10.1016/S0161-6420(90)32483-1, 2-s2.0-0025313105, 2381705. 2381705

