# Supplementary material for: A Case of Refractory Childhood Glaucoma Associated With Sturge–Weber Syndrome Treated With Baerveldt Glaucoma Implant
Source: Case Rep Ophthalmol Med. 2026 Jan 8;2026:8624998. doi: 10.1155/crop/8624998 (PMC12781999; doi:10.1155/crop/8624998)
Supplement: Supplementary file 1 — Supporting Information 1 Additional supporting information can be found online in the Supporting Information section. Figure S1: OCT image of his right eye (OD) shows normal choroidal vascular pattern, whereas his left eye (OS) shows increased choroidal thickness and loss of choroidal vascular pattern. [file CROP-2026-8624998-s001.pdf]

Supplementary Figure 1

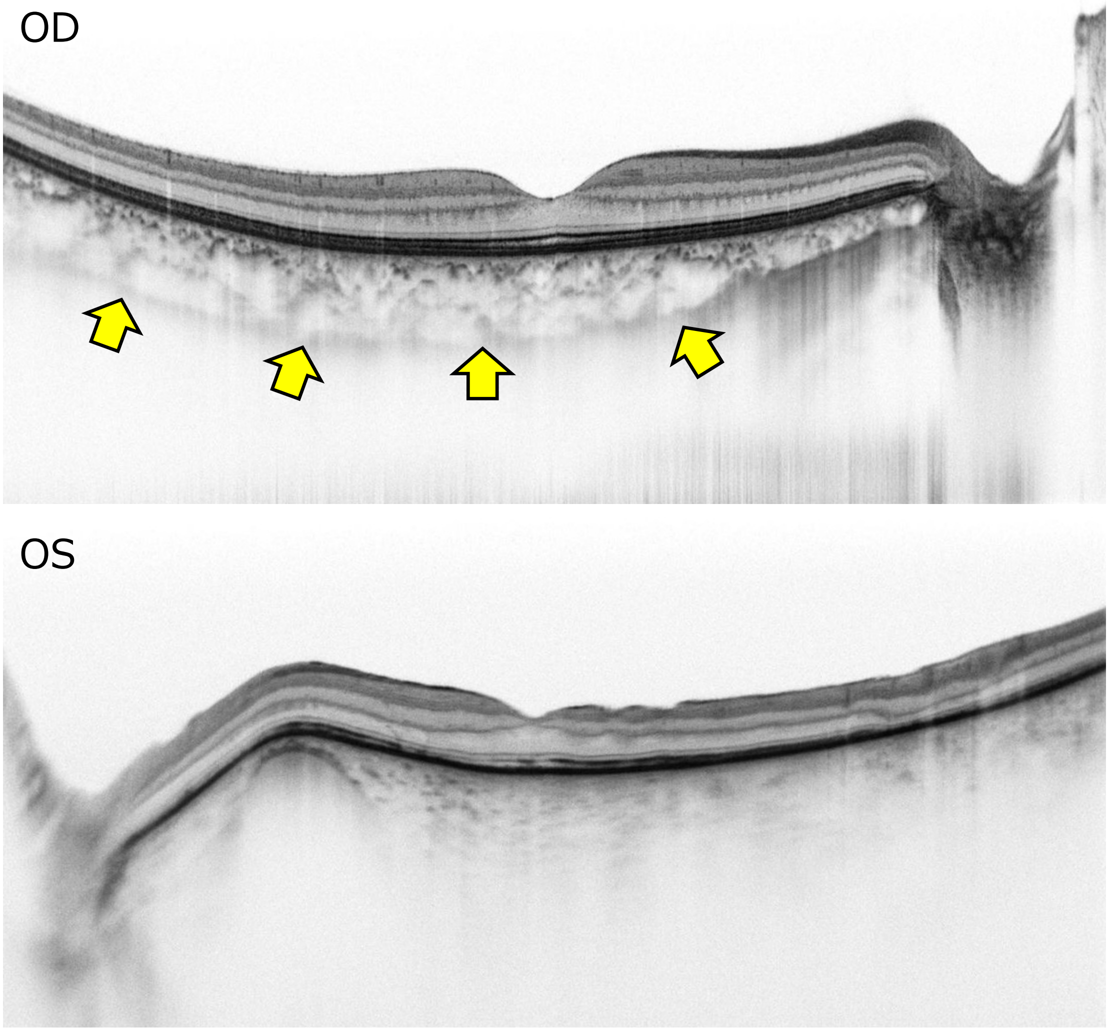

OCT image of his right eye (OD) shows normal choroidal vascular pattern, whereas his left eye (OS) shows increased choroidal thickness and loss of choroidal vascular pattern.
